# Pyruvate attenuates the anti-neoplastic effect of carnosine independently from oxidative phosphorylation

**DOI:** 10.18632/oncotarget.13039

**Published:** 2016-11-03

**Authors:** Henry Oppermann, Lutz Schnabel, Jürgen Meixensberger, Frank Gaunitz

**Affiliations:** ^1^ Klinik und Poliklinik für Neurochirurgie, Universitätsklinikum Leipzig AöR, 04103 Leipzig, Germany

**Keywords:** glioblastoma, carnosine, glycolysis, glucose, pyruvate

## Abstract

Here we analyzed whether the anti-neoplastic effect of carnosine, which inhibits glycolytic ATP production, can be antagonized by ATP production via oxidative phosphorylation fueled by pyruvate. Therefore, glioblastoma cells were cultivated in medium supplemented with glucose, galactose or pyruvate and in the presence or absence of carnosine. CPI-613 was employed to inhibit the entry of pyruvate into the tricarboxylic acid cycle and 2,4-dinitrophenol to inhibit oxidative phosphorylation. Energy metabolism and viability were assessed by cell based assays and histochemistry.

ATP in cell lysates and dehydrogenase activity in living cells revealed a strong reduction of viability under the influence of carnosine when cells received glucose or galactose but not in the presence of pyruvate. CPI-613 and 2,4-dinitrophenol reduced viability of cells cultivated in pyruvate, but no effect was seen in the presence of glucose. No effect of carnosine on viability was observed in the presence of glucose and pyruvate even in the presence of 2,4-dinitrophenol or CPI-613.

In conclusion, glioblastoma cells produce ATP from pyruvate via the tricarboxylic acid cycle and oxidative phosphorylation in the absence of a glycolytic substrate. In addition, pyruvate attenuates the anti-neoplastic effect of carnosine, even when ATP production via tricarboxylic acid cycle and oxidative phosphorylation is blocked. We also observed an inhibitory effect of carnosine on the tricarboxylic acid cycle and a stimulating effect of 2,4-dinitrophenol on glycolytic ATP production.

## INTRODUCTION

The anti-neoplastic effect of carnosine has been described for a number of tumor derived cells *in vitro* including gastric [1, 2], colon [3], ovarian [4] and brain cancer cells [5]. In addition, effects were demonstrated *in vivo* [6, 7] and the number of examples is still increasing (for reviews see [8, 9, 10]). The primary molecular targets responsible for carnosine's action on tumor cells are still not known. Although, its influence on glycolytic ATP production, recognized to be crucial for tumor cell energy metabolism, has been suggested by previous experiments [11]. The dependence of tumor cells on glycolysis is known as the so-called Warburg effect. It describes that ATP production in cancer cells is frequently dependent on glycolysis resulting in the production of lactate even in the presence of oxygen. In normoxic conditions non-tumor cells produce ATP by oxidative phosphorylation (OxPhos) using reduction equivalents derived from the metabolization of pyruvate entering the tricarboxylic acid (TCA) cycle (for reviews see [12, 13]). The Warburg effect has originally been attributed to defects in the mitochondria of cancer cells. According to current knowledge this only holds true for a minority of tumors [14]. More recent data point towards variants of glycolytic enzymes that may specifically be expressed in tumors such as pyruvate kinase M2 [15]. Unfortunately, this knowledge has up to now not resulted in the development of new therapeutic strategies to fight cancer. Thus, a thorough investigation of the inhibitory effect of carnosine on tumor cell specific ATP production will greatly help to develop new strategies which can exploit the Warburg effect. This is especially pertinent for malignancies, for those chances of recovery are poor under present-day treatment strategies. Tumor cells may adapt to changes in nutritional supply by switching metabolic fluxes and/or become fed by compounds supplied by neighbor cells [16]. Hence a possible inhibition of glycolysis, attenuated by metabolic adaptation, has to be taken into account (for recent reviews see [17, 18]). More than 20 years ago, Holiday and McFarland suggested that carnosine's anti-neoplastic effect might be inhibited by the presence of pyruvate [19]. As carnosine inhibits glycolytic ATP production [11] the most straight interpretation of the observation of Holiday and McFarland would be a tumor cell switch to OxPhos when glycolysis is inhibited and pyruvate is supplied. Therefore, we analyzed the response of tumor cell viability measuring ATP in cell lysates and dehydrogenase activities (NAD(P)H) in living cells. We used cells from human glioblastoma (GBM) which is the most common primary tumor of the adult brain [20]. According to the classification of the world health organization (WHO), GBM is one of the most malignant diffuse astrocytic tumors and classified as WHO grade IV [21]. Currently, the median overall survival of patients receiving standard therapy after surgery of the tumor is 14.6 month [22]. Consequently, there is urgent need to develop alternative treatment strategies. These may include a metabolic intervention at the level of glycolysis as glucose is the central metabolic fuel of this tumor. Our experiments were mainly performed with cells cultivated in the presence of glucose. We also tested galactose as a nutritional substitute for glucose in a first series of experiments. The cells were cultivated in the absence and presence of carnosine and we analyzed the influence of pyruvate on carnosine's anti-neoplastic effect. In order to determine the influence of the TCA cycle and of OxPhos the experiments were also performed in the absence and presence of inhibitors for the pyruvate dehydrogenase complex and for ATP production by OxPhos. In addition, we established a protocol in which the cells were pre-starved in the absence of glucose, glutamine and serum. Effects from the presence of compounds the cells were exposed to during long term cultivation were thus avoided. This appeared to be especially important with regard to serum that was omitted throughout the experiments because it contains compounds of undefined nature.

## RESULTS

### Viability, amount of ATP and NAD(P)H production in glioblastoma cells cultivated in glucose, galactose or pyruvate under the influence of serum and GlutaMax

In previous experiments, in which the anti-neoplastic effect of carnosine on glioblastoma cells was analyzed, a high variation of viability was encountered [23]. Comparing eight independent experiments, in which the effect of 50 mM carnosine in U87 cells after 24 hours of incubation was determined, we found a reduction of ATP in cell lysates between 44% and 89% (75 ± 15%; data not shown) compared to the untreated control. In these experiments cells received medium supplemented with carnosine after they had been grown in medium with 4.5 g/L glucose, 10% FBS and 2 mM GlutaMax. Therefore, it cannot be ruled out that the effect of carnosine on ATP production was concealed by intracellular metabolites, contributing to ATP production, downstream of carnosine's target.

**Figure 1:**
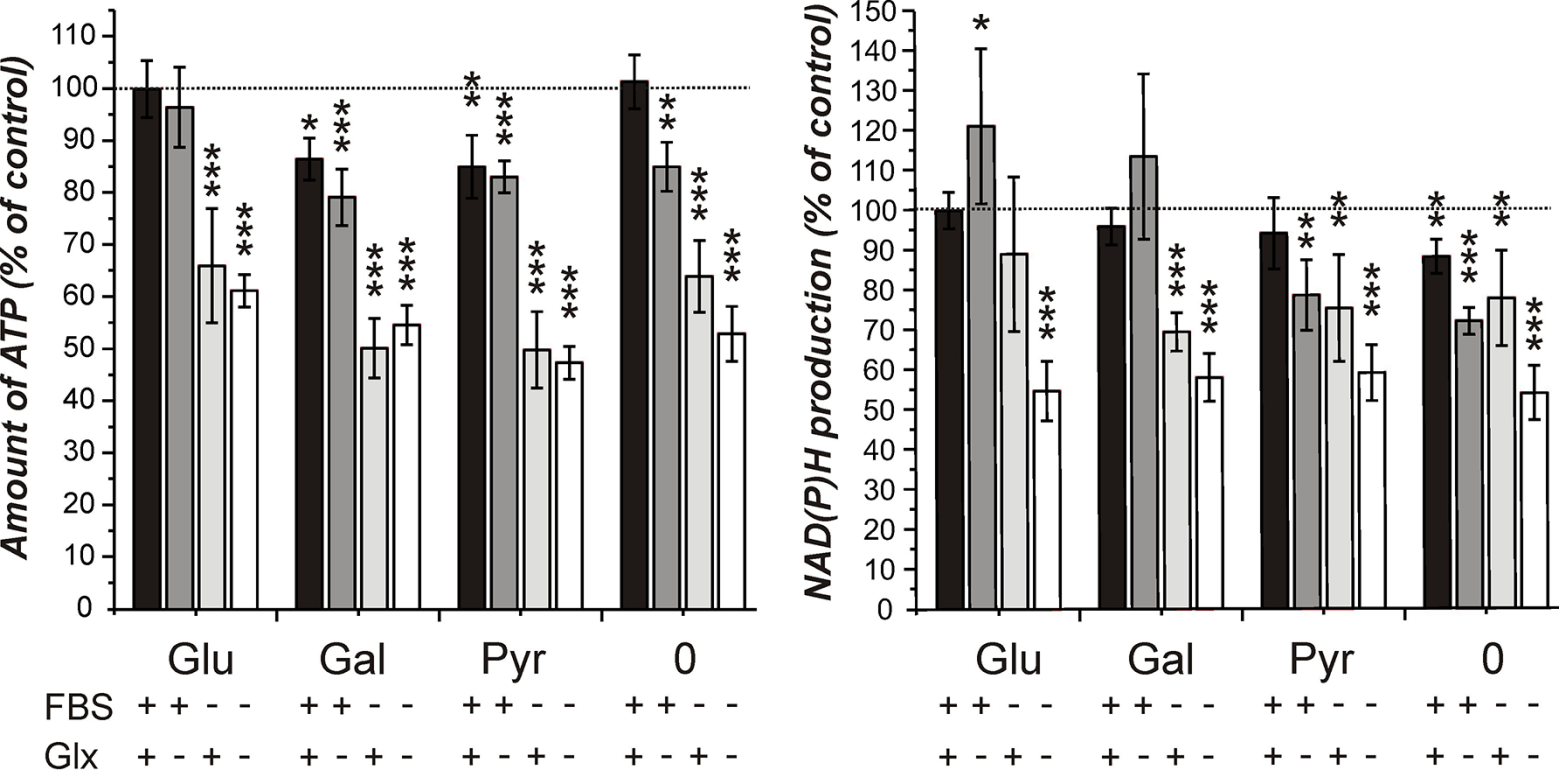
Amount of ATP in cell lysates and NAD(P)H production in cells from the line U87 under different culture conditions Cells from the line U87 were cultivated for 20 hours at a density of 5000 cells per well in 96-well microplates in DMEM standard culture medium before they received fresh medium with different supplements: FBS: fetal bovine serum, 10%; Glx: GlutaMax, 2 mM; Glu/Gal/Pyr: glucose, galactose, pyruvate: each 25 mM; 0: no Glu/Gal/Pyr. 24 hours later the CellTiter-Glo assay (measuring ATP in cell lysates, left part of the panel) and the CellTiter-Blue assay (measuring NAD(P)H production, right part of the panel) were employed to assess cell viability. Signals obtained from cells cultivated in 25 mM glucose, 10% FBS and 2 mM GlutaMax were set as 100%. Results are presented as mean and standard deviation of 6 wells. Statistical significance was determined by Student's *t-test* with: *p < 0.05; **p < 0.005; ***p < 0.0005.

This question was addressed by firstly investigating the viability of cells cultivated for 24 hours in medium without the supplements glucose, GlutaMax and FBS. The result of one representative experiment is presented in Figure 1. In addition, we investigated whether galactose and pyruvate are able to substitute glucose. As can be seen in Figure 1, only the absence of FBS from the culture medium prominently decreased viability as revealed by reduced dehydrogenase activity and ATP in cell lysates. Additionally, no signs of apoptosis or necrosis were observed. Galactose as well as pyruvate could substitute glucose to a large extent. More importantly, the viability of cells that were cultivated in the absence of glucose, galactose or pyruvate did not severely differ from that of cells cultivated in the presence of the nutrients or did exhibit morphological change (data not shown). The experiment indicates an influence of FBS on viability and demonstrates that a 24 hour starvation in the absence of glucose, galactose or pyruvate had only minor effects on viability. The determination of lactate dehydrogenase release into the medium, as a marker of necrotic loss of membrane integrity, did not reveal cell death by necrosis (data not shown). Further experiments with cells cultivated without glucose, galactose or pyruvate and without FBS and GlutaMax prior to the addition of carnosine were hence decided.

### Viability, amount of ATP and NAD(P)H production of pre-starved glioblastoma cells under the influence of carnosine and different nutrients

**Figure 2:**
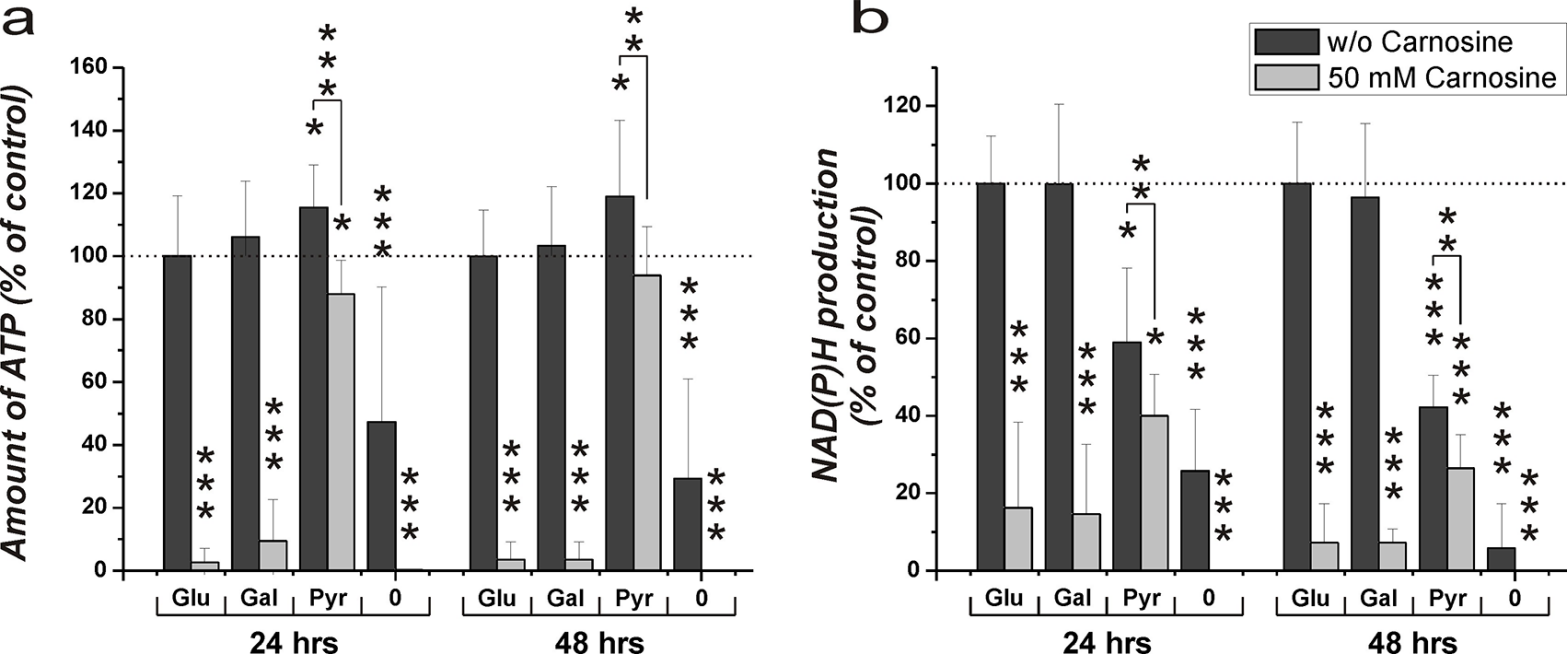
Amount of ATP in cell lysates and NAD(P)H production in the line U87 under the influence of glucose, galactose or pyruvate and carnosine after pre-starvation Cells from the line U87 were seeded at a density of 5000 cells per well in 96-well microplates and were incubated for 20 hours in medium without glucose, galactose or pyruvate and without FBS and GlutaMax. After 20 hours, medium was substituted for medium with different supplements: glucose (Glu), galactose (Gal), pyruvate (Pyr): 25 mM, no supplement (0); and with or without 50 mM carnosine. 24 and 48 hours later CellTiter-Glo and CellTiter-Blue assays were employed to determine ATP in cell lysates and NAD(P)H production in intact cells. In order to compare the effects of glucose, galactose and pyruvate in the presence and absence of carnosine, results are grouped into data sets with regard to the amount of ATP in cell lysates (**A**) and dehydrogenase activity (**B**). Results are represented as mean and standard deviation of 6 wells from three independent experiments for each condition normalized to signals recorded from cells cultivated in the presence of glucose and the absence of carnosine (set as 100%). Statistical significance was determined by Student's *t-test* with: *p < 0.05; **p < 0.005; ***p < 0.0005.

The cultivation of cells for 24 hours in the absence of FBS, GlutaMax and the specified nutrients did not result in a pronounced reduction of viability. Therefore, experiments with carnosine were performed with cells pre-cultivated for 20 hours in the absence of the aforementioned supplements. This avoids effects which could flaw the experiments as described in the introduction. Cells from the glioblastoma cell line U87 were transferred to 96-well plates at a density of 5000 cells per well, seeded for 3 hours in fully supplemented medium and then cultivated in the absence of glucose, galactose or pyruvate and without FBS and GlutaMax for 20 hours. Then, fresh medium was added containing glucose, galactose, pyruvate or without any of these compounds and supplemented with or without 50 mM carnosine. Twenty-four and forty-eight hours later viability was determined by measuring ATP in cell lysates and dehydrogenase activity in living cells. As can be seen in Figure 2A, cells cultivated in glucose, galactose and pyruvate exhibited a comparable amount of ATP in cell lysates after 24 and 48 hours in the absence of carnosine. Whereas carnosine strongly reduced viability in cells cultivated in the presence of glucose and galactose (down to 3.6 ± 5.6% at 48 hours), but not in those cells incubated in the presence of pyruvate (94.0 ± 15.5% at 48 hours). This demonstrates strong inhibition of the glycolytic ATP production by carnosine. In addition, it indicates that the tumor cells can compensate insufficient production of ATP by glycolysis by fueling pyruvate into the TCA cycle resulting in ATP production by OxPhos. As there was a distinctively measurable amount of ATP in cell lysates of cells cultivated without any supplement and in the absence of carnosine (47.3 ± 42.9% at 24 hours + 20 hours of starvation and 29.3 ± 31.7% at 48 hours + 20 hours of starvation compared to cells in the presence of glucose), but not in the presence of carnosine (0.2 ± 0.1% at 44 and 68 hours of total starvation time), this ATP may be generated from metabolites that are present even after 44 and 68 hours of starvation. This ATP production, however, is clearly inhibited by carnosine. Comparing the production of NAD(P)H in living cells (Figure 2B) it becomes obvious that pyruvate does not completely substitute galactose or glucose. The glycolytic production of redox equivalents appears to be higher than that from pyruvate, but this may be caused by a rapid mitochondrial metabolization of TCA cycle derived redox equivalents. The minor difference between production of NAD(P)H in medium containing pyruvate with or without carnosine may indicate that there are still metabolites present which account for the production of NAD(P)H. Moreover, this notion is substantiated by the observation that there is still some NAD(P)H production in medium containing either glucose or galactose and carnosine. It also has to be taken into account that the CellTiter-Blue assay also measures the production of reduced reduction equivalents from other dehydrogenases aside from the glyceraldehyde-3-phosphate dehydrogenase reaction. However, as the production of NAD(P)H is higher in medium containing pyruvate than in the medium without any supplement there is an obvious production of NAD(P)H via pyruvate. As revealed in Figure 7 and Table 1 no necrosis does occur in cells treated with carnosine in the presence of pyruvate.

**Table 1:** Cell viability in the presence of different nutritional compounds and inhibitors

	Glucose	Pyruvate	Glucose + Pyruvate
Control	94.30 ± 4.56%	98.54 ± 0.29%	98.63 ± 1.38%
Carnosine	0.00 ± 0.00%	99.25 ± 0.54%	95.58 ± 2.03%
CPI-613	93.79 ± 3.40%	27.41 ± 11.15%	99.18 ± 0.04%
Carnosine + CPI-613	0.47 ± 0.47%	2.72 ± 0.81%	98.37 ± 0.81%
DNP	97.13 ± 0.71%	21.06 ± 3.03%	96.48 ± 3.84%
Carnosine + DNP	1.65 ± 2.32%	1.95 ± 2.29%	98.03 ± 1.34%

Number of viable cells in the presence of different nutrients and inhibitors as determined by Calcein AM/PI staining from the experiments depicted in Figure 5 to 7 expressed in % of cells stained with Calcein AM compared to the total number of cells.

### Viability and the amount of ATP in glioblastoma cells in the presence of the pyruvate dehydrogenase inhibitor CPI-613

**Figure 3:**
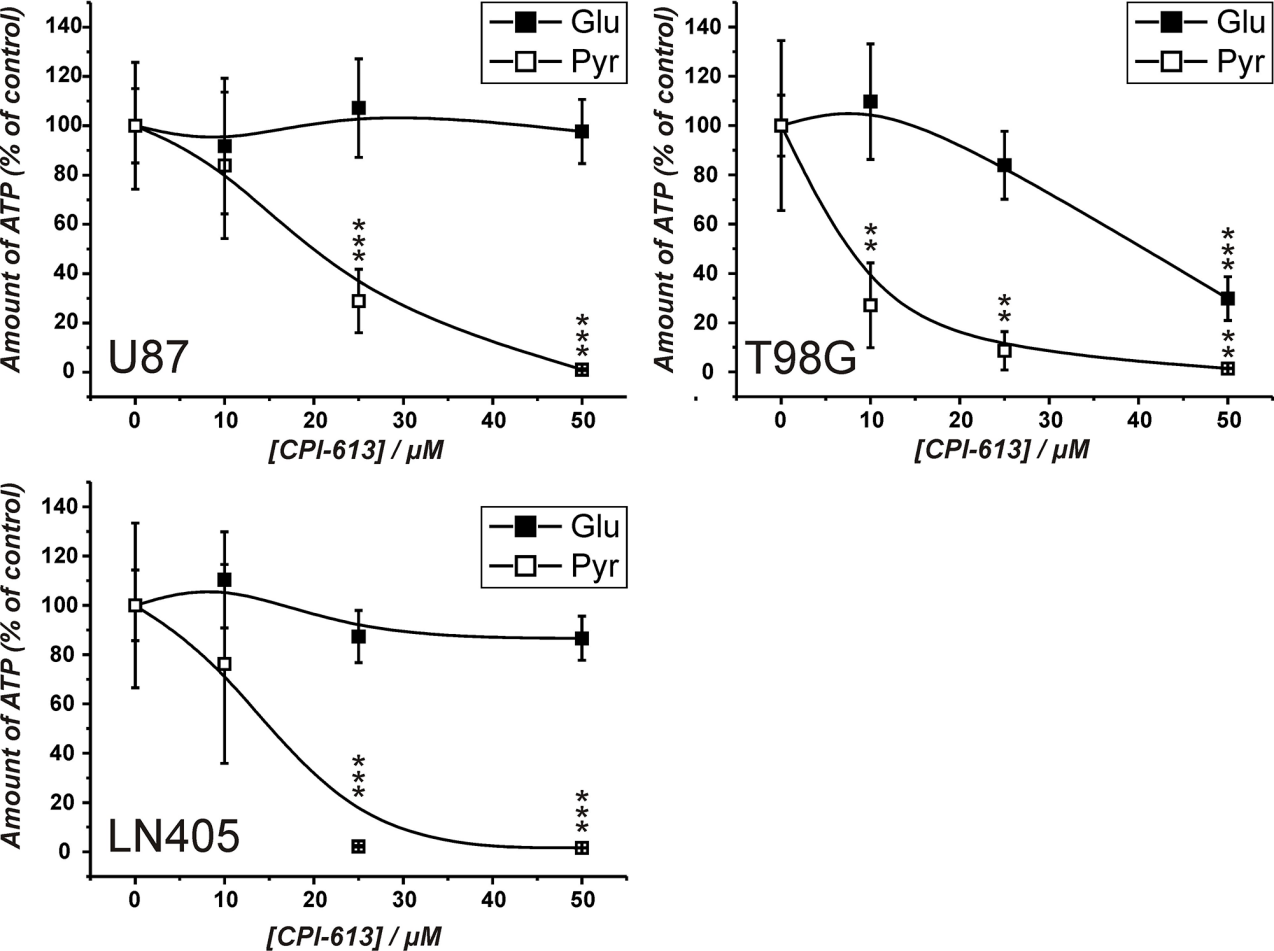
Amount of ATP in cell lysates of cells from the lines U87, T98G and LN405 under the influence of the pyruvate dehydrogenase inhibitor CPI-613 Cells were seeded at a density of 5000 cells per well in 96-well microplates before they received medium without glucose, galactose or pyruvate and without FBS and GlutaMax. 20 hours later fresh medium was added with different concentrations of CPI-613 (0 μM, 10 μM, 25 μM, 50 μM) in the presence of either glucose (Glu; 25 mM) or pyruvate (Pyr; 5 mM). 24 hours later ATP amount in cell lysates was determined using the CellTiter-Glo assay. ATP amount in cell lysates in the absence of inhibitor was set as 100%. Results are represented as mean and standard deviation of 6 wells for each condition. Statistical significance was determined by Student's *t-test* with: **p < 0.005 and ***p < 0.0005.

The experiments presented in Figure 2 demonstrate that cells from the line U87 are viable in the absence of a glycolytic substrate, when pyruvate is present. This indicates that the cells can produce ATP from pyruvate via the TCA cycle and OxPhos in the absence of glucose, which we just recently demonstrated by the analysis of metabolic profiles, determined by gas chromatography coupled to mass spectrometry (GC-MS) [24]. Hence, it was asked whether the flux of metabolites through the TCA cycle can antagonize the effect of carnosine on ATP production. Therefore, the pyruvate dehydrogenase inhibitor CPI-613 was used to block the initial step that converts pyruvate to acetyl-CoA fueling the TCA cycle [25]. CPI-613 is a lipoic acid analog which activates the lipoate-responsive regulatory phosphorylation of the E1α pyruvate dehydrogenase subunit and was shown to be selective for tumor cells in culture [25]. The experiments were performed with cells from the glioblastoma lines LN405 and T98G in addition to U87 in order to analyze a cell line dependent difference. For the experiment, cells from the three lines were pre-starved for 20 hours as described above, before they received medium with either 25 mM glucose or 5 mM pyruvate and different concentrations of CPI-613 for 24 hours. A concentration of 5 mM pyruvate was chosen, as experiments revealed that this concentration is sufficient for the maximum amount of ATP determined in cell lysates in the presence of 50 mM carnosine (Supplementary Data S1). As shown in Figure 3, the amount of ATP in cell lysates in the presence of CPI-613 was progressively reduced with increasing concentrations of the inhibitor when pyruvate was supplied as nutrient. This supports the notion that pyruvate can be metabolized by the TCA cycle and that OxPhos is not impaired in these cells. Furthermore, cells from the lines U87 and LN405 did not show any significant change in the amount of ATP in the presence of glucose and CPI-613, whereas ATP of cells from the line T98G was strongly reduced. This demonstrates that T98G cells are dependent on the TCA cycle and OxPhos. At this point it is interesting to note, that in the absence of glucose, pyruvate does differently contribute to the amount of ATP (in the absence of the inhibitor) in the three lines (data not shown in the graph): Whereas ATP in U87 and T98G cells is not significantly different between cells either supplied with glucose or pyruvate (glucose to pyruvate in U87: 100 ± 15.1% and 120.2 ± 31%, in T98G: 100 ± 12.4% and 83 ± 28.6%), pyruvate does less efficiently contribute to ATP in cells from the line LN405 (glucose to pyruvate: 100 ± 14.3% and 39 ± 13%; *p* < 0.005).

### Viability and amount of ATP in glioblastoma cells under the influence of carnosine in the presence of glucose and glucose plus pyruvate

**Figure 4:**
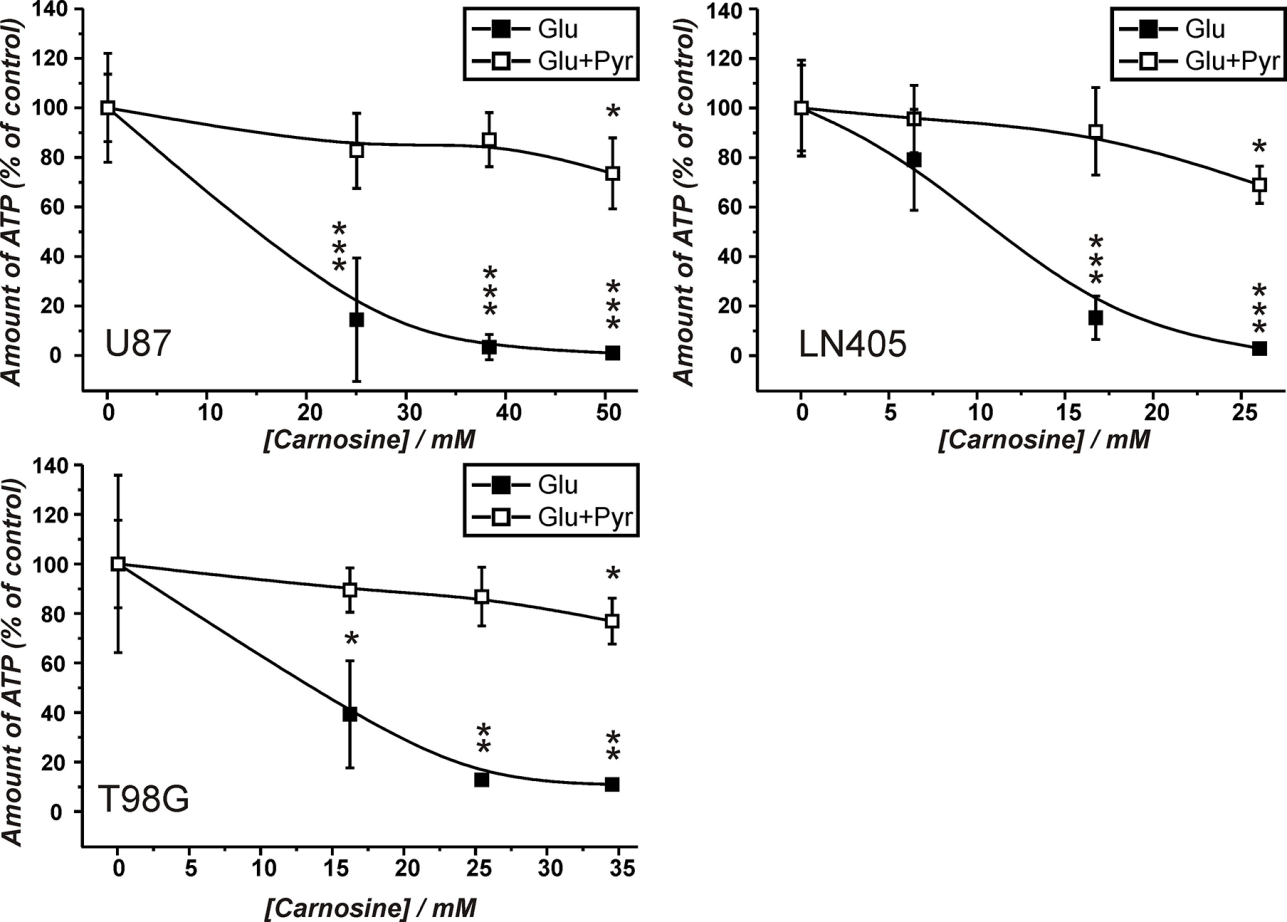
Amount of ATP in cell lysates of the lines U87, T98G and LN405 under the influence of different concentrations of carnosine in the presence of glucose and glucose plus pyruvate Cells were seeded at a density of 5000 cells per well in 96-well microplates and received medium without glucose, galactose or pyruvate and without FBS and GlutaMax. After 20 hours, fresh medium was added with either glucose (Glu; 25 mM) or a mixture of 25 mM glucose and 5 mM pyruvate (Glu+Pyr) and cell line dependent inhibitory concentrations of carnosine (see Supplementary Data S2). 24 hours later the amount of ATP in cell lysates was determined using the CellTiter-Glo assay. The amount of ATP in the absence of carnosine was set as 100%. Results are represented as mean and standard deviation of 6 wells for each condition. Statistical significance was determined by Student's *t-test* with: *p < 0.05; **p < 0.005; ***p < 0.0005.

The experiments in the previous sections demonstrated that the production of ATP via pyruvate is possible in glioblastoma cells (although not required when glucose is abundant - at least in cells from the lines U87 and LN405), but may become important in the absence of glucose. To consider using carnosine's capacity to block the glycolytic flux from glucose in tumor cells for the treatment of human tumors, it had to be asked whether the anti-neoplastic effect of the dipeptide may be attenuated by pyruvate. Therefore, cells from the glioblastoma lines U87, LN405 and T98G were incubated in the presence of either glucose (25 mM) or a mixture of glucose (25 mM) and pyruvate (5 mM) and in the absence and presence of different concentrations of carnosine determined to equal an inhibitory concentration of IC20, IC50 and IC80 (see Supplementary Data S2). The data presented in Figure 4 demonstrates that in all three lines the inhibitory effect of carnosine on cell viability in the presence of glucose is strongly attenuated by the addition of pyruvate. Only at the highest concentration of carnosine (IC80) a significantly reduced amount of ATP in the presence of glucose and pyruvate is observed. In fact, cells from the line U87 grown in the presence of glucose and 50 mM carnosine exhibit an almost complete loss of viability after a 24 hour exposure to the dipeptide. Cells, which received pyruvate in addition, were as healthy as cells cultivated in the absence of carnosine (Figure 7; Table 1). At this point it also has to be noted that the amount of ATP in the presence of glucose and pyruvate compared to glucose alone was ∼2-fold higher in LN405 and comparable in U87 cells (1.3-fold higher). The amount of ATP in cells from the line T98G was ∼10-fold higher in the presence of pyruvate and glucose compared to glucose alone. This underscores that ATP production from pyruvate via the TCA cycle significantly contributes to ATP production in cells from the line T98G, but also demonstrates that ATP can also be produced by OxPhos in the other cell lines. Comparing this data to the results in the preceding section, this reveals a different response of cells to the supply with nutrients. Cells from the line T98G exhibit much higher amounts of ATP when both glucose and pyruvate are supplied than with either nutrient alone. This indicates that in these cells excessive ATP production by OxPhos also drives glycolytic ATP production - most likely via regeneration of NAD^+^, resulting in an overall synergistic effect on total ATP production in these cells.

### Amount of ATP and viability of U87 glioblastoma cells in the presence of glucose and pyruvate under the influence of carnosine and CPI-613

**Figure 5:**
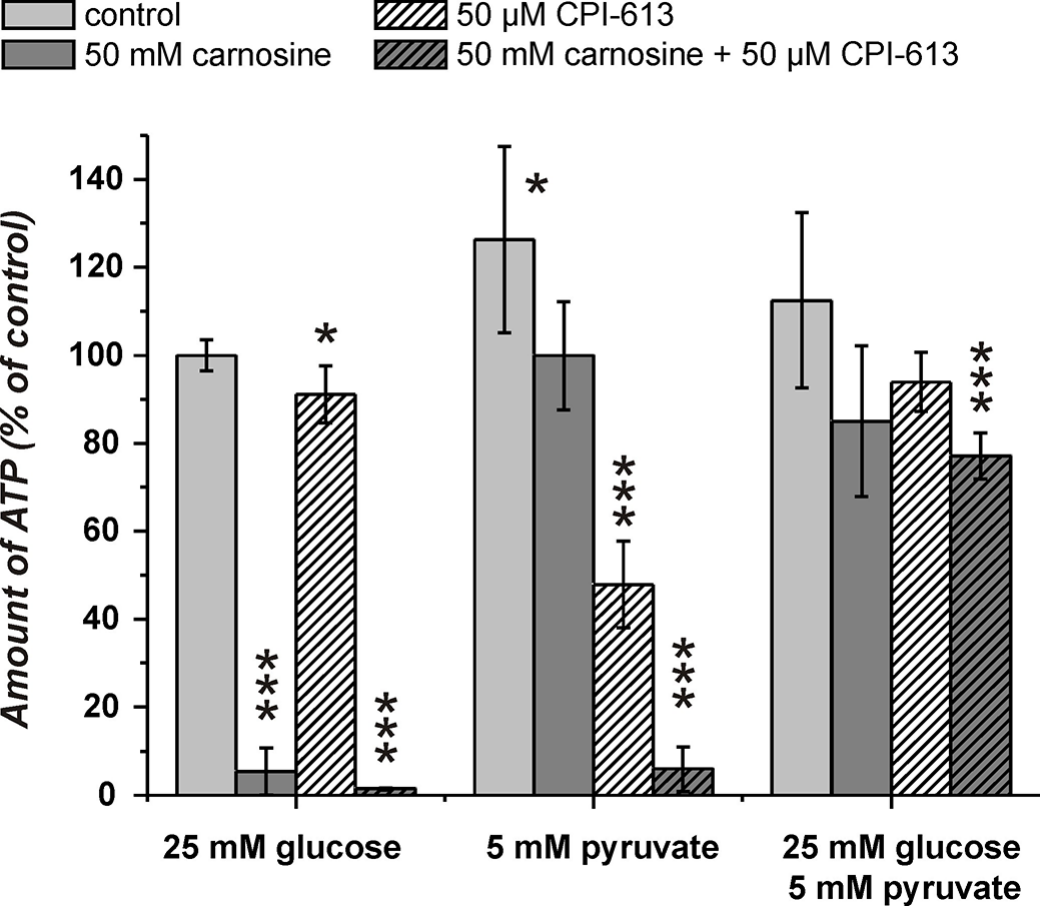
Amount of ATP in cell lysates of the line U87 under the influence of carnosine and CPI-613 Cells from the line U87 were seeded at a density of 5000 cells per well in 96-well microplates and received medium without glucose, galactose or pyruvate and without GlutaMax and FBS for 20 hours. Then, fresh medium was added with 25 mM glucose, 5 mM pyruvate or a combination of both nutrients. In addition, cells received 50 mM carnosine and/or 50 μM CPI-613. 24 hours later the amount of ATP in cell lysates was determined. Results are represented as mean and standard deviation of 6 wells for each condition normalized to cells cultivated in the presence of glucose (set as 100%). Statistical significance was determined by Student's *t-test* with: *p < 0.05; ***p < 0.0005.

The experiments presented in Figure 4 indicated that pyruvate attenuates the inhibitory effect of carnosine on ATP production and viability in cells cultivated in glucose alone. As ATP production from pyruvate requires its entry into the TCA cycle, we asked whether the combined block of glycolysis by carnosine and of pyruvate dehydrogenase by CPI-613 does completely block ATP production in U87 cells. The result of a corresponding experiment is presented in Figure 5. This experiment demonstrates that the combination of carnosine and CPI-613 almost completely blocks ATP production in the presence of either glucose (1.5 ± 0.2%) or pyruvate alone (5.9 ± 5.1%). The viability of cells as determined by Calcein AM/PI staining was reduced to 0.5 ± 0.5% (glucose) and to 2.7 ± 0.8% (pyruvate) (see Figure 7 and Table 1). CPI-613 alone did only slightly reduce the amount of ATP determined in the presence of glucose (91.1 ± 6.5%; *p* < 0.05) and viability determined by Calcein AM/PI was 93.8 ± 3.4% compared to 94.3 ± 4.6% in the absence of CPI-613. When cells cultivated in pyruvate received CPI-613 alone, the amount of ATP was reduced from 126 ± 21% to 48 ± 10% and cell viability determined by Calcein AM/PI was reduced from 98.5 ± 0.3% to 27.4 ± 11.2% (*p* < 0.02; Figure 7; Table 1). Therefore, the cells were still able to produce some ATP and were not completely killed during the 24 hours incubation time. Most importantly, although viability was strongly reduced when only glucose or only pyruvate were supplied in the presence of carnosine and CPI-613, viability in the presence of both, glucose and pyruvate, was completely restored (98.4 ± 0.8%). Correspondingly, ATP production was restored, although significantly lower amounts were detected (77.1 ± 5.3%; *p* < 0.0005) than in cells cultivated in the presence of glucose and pyruvate without the inhibitors (112.5 ± 19.9%).

### Viability and amount of ATP in U87 glioblastoma cells in the presence of glucose and pyruvate under the influence of carnosine and 2,4-dinitrophenol

**Figure 6:**
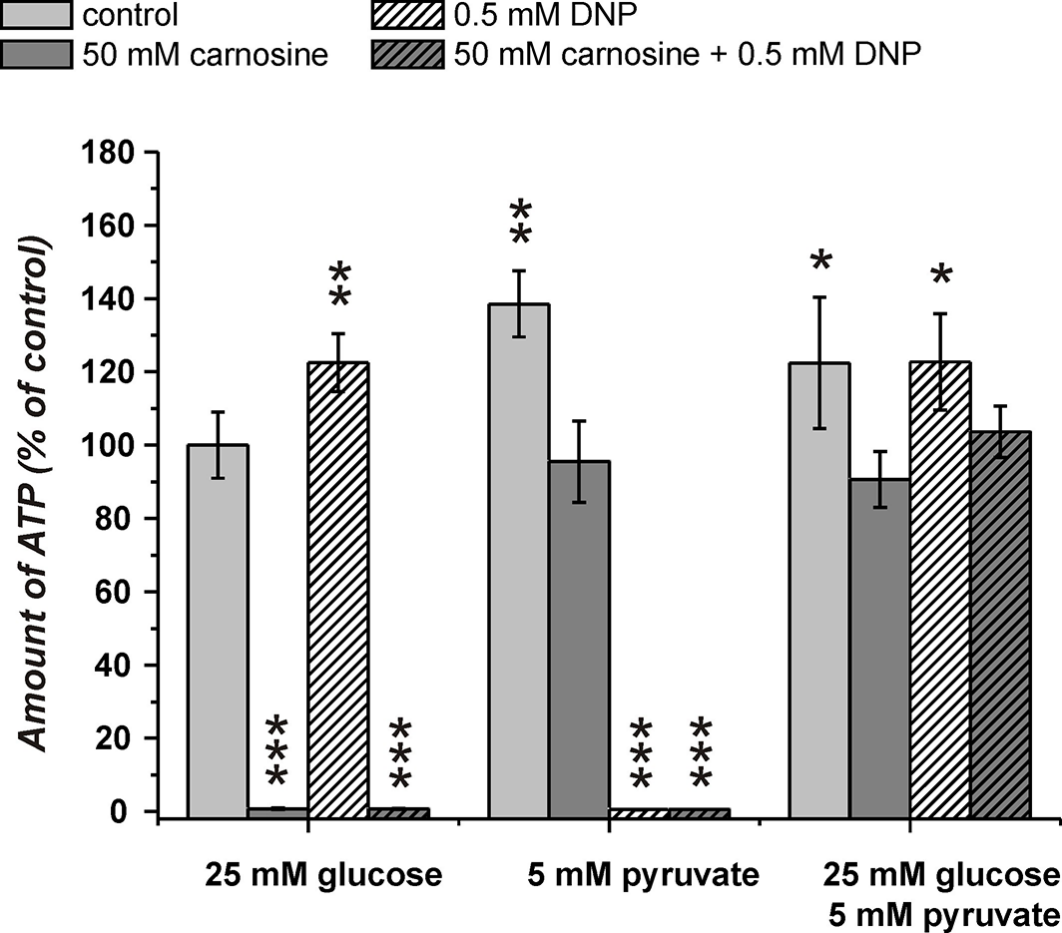
Amount of ATP in cell lysates of the line U87 under the influence of carnosine and 2,4-dinitrophenol (DNP) Cells from the line U87 were seeded at a density of 5000 cells per well in 96-well microplates and received medium without glucose, galactose or pyruvate and without GlutaMax and FBS for 20 hours. Then, fresh medium was added with 25 mM glucose, 5 mM pyruvate or a combination of both nutrients. In addition, cells received 50 mM carnosine and/or 0.5 mM DNP. 24 hours later the amount of ATP in cell lysates was determined. Results are represented as mean and standard deviation of 6 wells for each condition normalized to cells cultivated in the presence of glucose (set as 100%). Statistical significance was determined by Student's *t-test* with: *p < 0.05; **p < 0.005; ***p < 0.0005.

**Figure 7:**
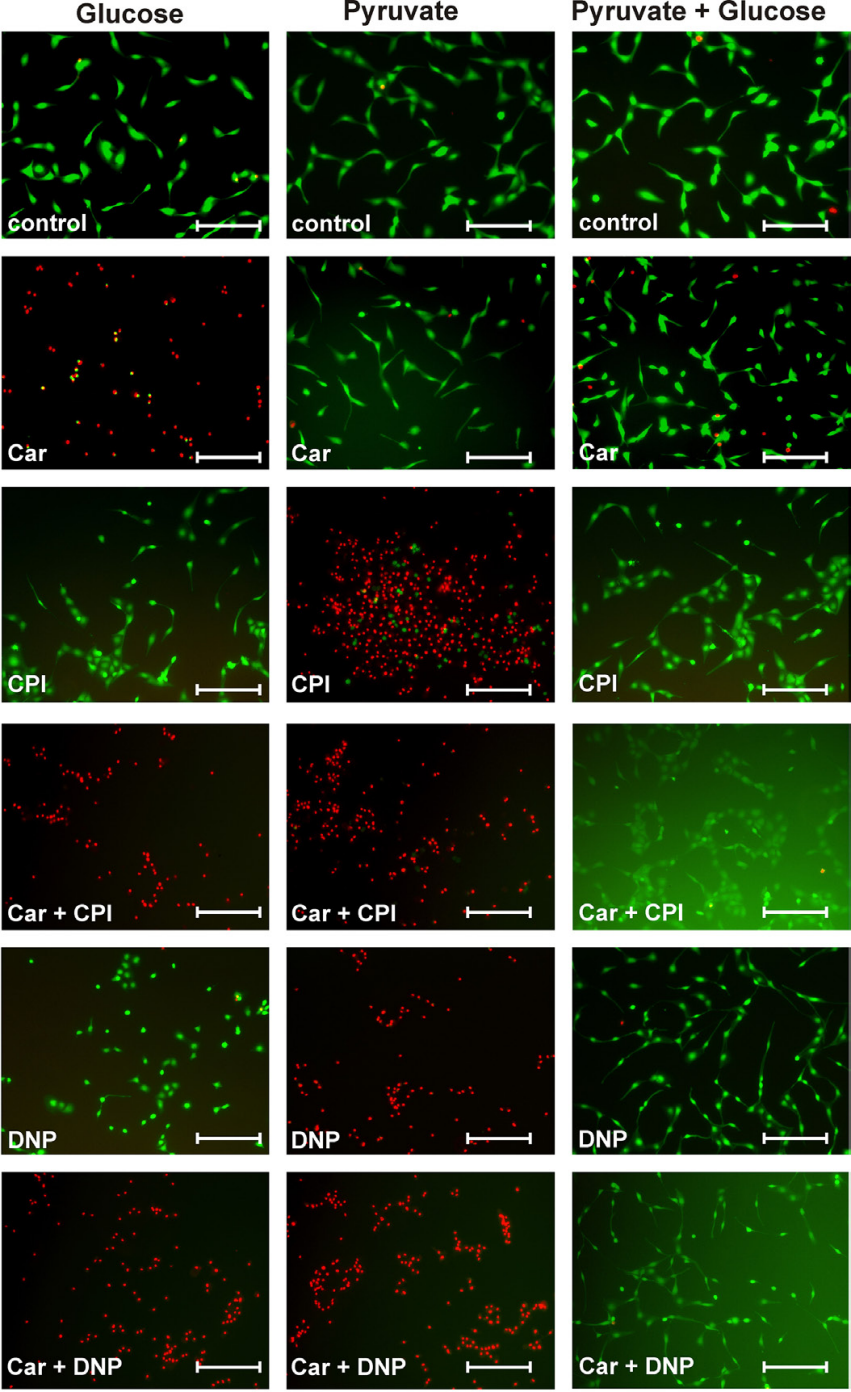
Viability of U87 cells under the influence of carnosine in the presence of glucose, pyruvate, carnosine (Car), 2,4-dinitrophenol (DNP) and CPI-613 (CPI) U87 cells were seeded at a density of 5000 cells per well in 96-well microplates and received medium without glucose, galactose or pyruvate and without FBS and GlutaMax. After 20 hours, fresh medium was added with either glucose (25 mM), pyruvate (5 mM) or both nutrients and in addition cells received carnosine (50 mM), CPI-613 (50 μM) or DNP (0.5 mM) or mixtures of these supplements. After 24 hours living cells were stained using Calcein AM (green) and dead cells were stained using propidium iodide (red). Fluorescence images were captured by microscopy. Scale bars: 200 μm. For quantification see Table 1.

The experiments in the previous section revealed that the combination of carnosine and CPI-613 in the presence of pyruvate and glucose does not kill the cells. This observation is in contrast to the notion that pyruvate's attenuation of the inhibitory effect of carnosine is dependent on ATP production from pyruvate via TCA cycle and OxPhos. However, as we observed viable cells in the presence of pyruvate alone and CPI-613 (27.4 ± 11.2%) and also a significant amount of ATP in cell lysates (48 ± 10%) (Figure 5), we decided to block ATP production additionally at the level of OxPhos using 2,4-dinitrophenol (DNP). DNP inhibits mitochondrial ATP production by uncoupling electron transport from phosphorylation [26]. In the experiment in Figure 6 cells from the line U87 were incubated with combinations of carnosine, DNP, glucose and pyruvate. In cells fed with glucose, DNP did not reduce the amount of ATP (122 ± 7.9% compared to 100 ± 9% in cells without the inhibitor). The viability determined by Calcein AM/PI was 97.1 ± 0.7% compared to 94.3 ± 4.6% (without DNP) (Figure 7; Table 1). Cells fed with pyruvate exhibited a strong reduction of ATP when incubated in the presence of DNP (0.5 ± 0.1% compared to cells in the absence of the inhibitor 138.5 ± 9%). The viability determined by Calcein AM/PI was reduced from 98.5 ± 0.3% to 21.1 ± 3% (Figure 7; Table 1). This demonstrates that DNP completely blocks ATP production from pyruvate, but also shows that a fraction of cells is able to survive at least after 24 hours of exposure to DNP. Therefore, it is tempting to speculate that survival of some cells is independent from ATP production. Cells cultivated in glucose only exhibited a strong reduction of ATP when cultivated in carnosine (0.8 ± 0.2%; Figure 6) as already shown in Figure 5. Cells receiving pyruvate as nutrient exhibited a comparable amount of ATP in the presence of carnosine (95.5 ± 11.1%) as cells cultivated in glucose without it (100 ± 9.1%). As also seen in Figure 5 and Figure 2A, cells receiving pyruvate produced more ATP in the absence of inhibitors than cells cultivated in glucose (138.5 ± 9%). As the addition of carnosine always reduces ATP production in the presence of pyruvate alone, carnosine does obviously also inhibit mitochondrial ATP production, although to a lesser extent than it effects glycolytic ATP production. Most importantly, in the presence of both, pyruvate and glucose, the amount of ATP determined in the presence of carnosine and DNP (103.7 ± 7.0%) was comparable to that of cells grown in glucose without any inhibitor (100 ± 9.1%). In addition, viability determined by Calcein AM/PI was 98.0 ± 1.3% (Figure 7; Table 1). This demonstrates that the attenuation of the anti-neoplastic effect of carnosine by pyruvate is independent from the production of ATP via the TCA cycle and OxPhos.

## DISCUSSION

We investigated how the anti-neoplastic effect of carnosine is influenced by the nutritional supply of tumor cells and how glycolysis, the TCA cycle and OxPhos contribute to tumor cell survival. It was previously shown that pyruvate inhibits the anti-neoplastic effect of carnosine [19]. We were thus interested to study, whether this can also be seen in glioblastoma tumor cells and whether the production of ATP via TCA cycle and OxPhos are responsible for this effect.

**Figure 8:**
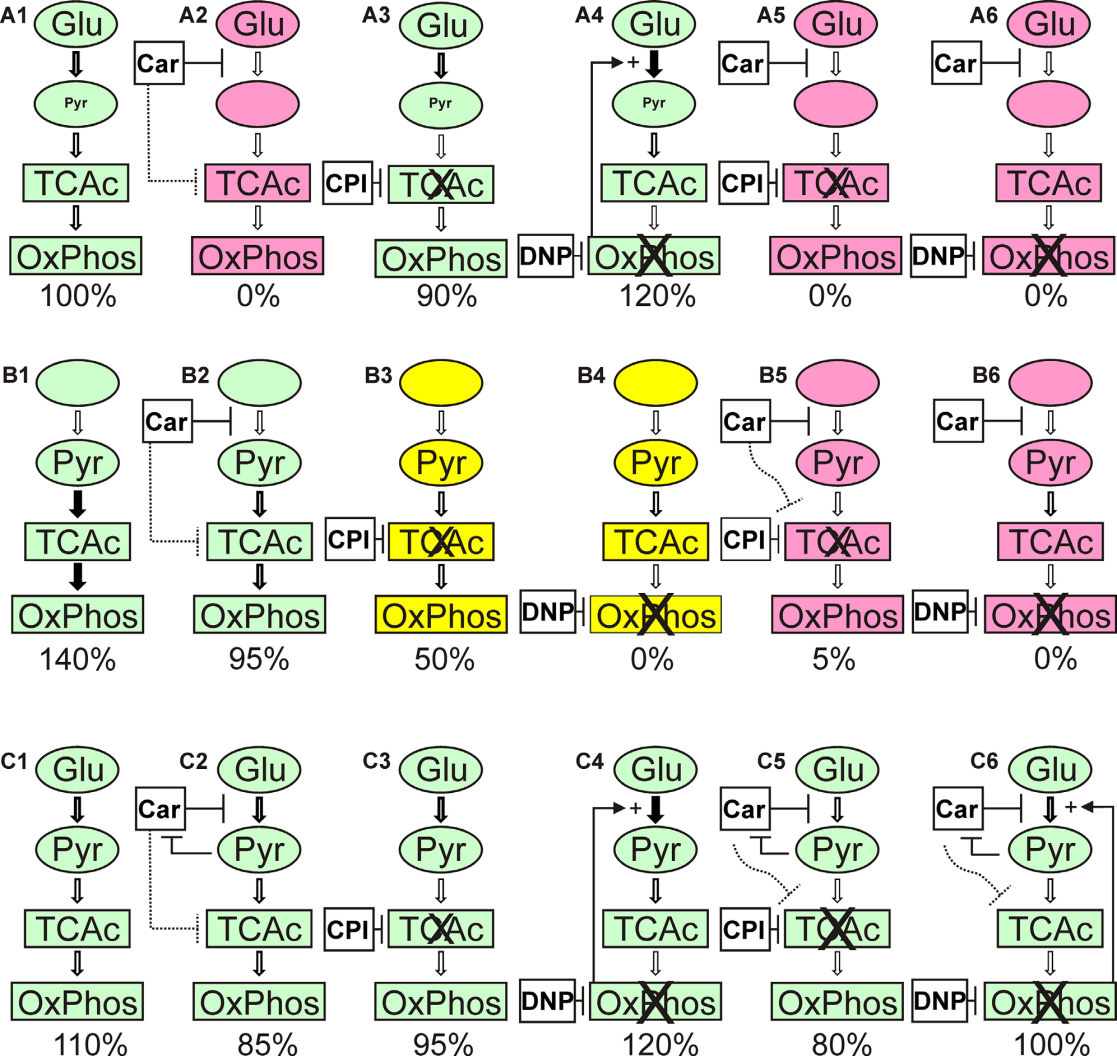
Overview of the results from the experiments presented in Figure 5 to Figure 7 and Table 1 A1 to A6 depict the experiments in the presence of glucose (Glu), B1 to B6 the experiments in the presence of pyruvate (Pyr) and C1 to C6 those with both, glucose and pyruvate. The numbers below denote the amount of ATP determined in cell lysates under each condition (rounded to be presented in intervals of 5). Colors denote: red: cells are dead; green: cells were alive; yellow: viable cells were detectable (20 to 30%; compare Table 1). Car denotes the use of carnosine, CPI denotes the use of CPI-613 and DNP the use of 2,4-dinitrophenol. The thickness of the arrows indicates the estimated flux of metabolites. For further details refer to the text.

The results of the experiments presented in Figure 5 to Figure 7 and in Table 1 are summarized and interpreted in Figure 8. In (A1) of Figure 8 the situation is depicted in which glucose is supplied as the only nutrient and in the absence of inhibitors. In this case the amount of ATP determined in cell lysates is set as 100% and living cells are illustrated by the green color. Adding carnosine (A2) results in a complete loss of ATP production and the cells are dead as shown by Calcein AM/PI staining. In accordance with our previously published data [5] no signs of apoptosis were detected and cells obviously died due to necrosis. Neither CPI-613 (A3) nor DNP (A4) decrease viability in the presence of glucose. Therefore, viability in the presence of glucose is almost independent from mitochondrial ATP production. However, adding CPI-613 to cells cultivated in glucose results in a small loss of ATP (A3 compared to A1), indicating that a small amount of pyruvate produced by glycolysis is used for mitochondrial ATP production. The slightly enhanced production of ATP in the presence of DNP (A4) demonstrates enhanced glycolytic production of ATP, when OxPhos is blocked by DNP. A comparable, recent observation by Bhatt *et al*. describes a rise of glycolytic ATP production in the cerebral glioma cell line BMG-1 and other cells when they were treated with DNP [27]. The addition of CPI-613 (A5) or DNP (A6) to cells in the presence of glucose and carnosine results in the same loss of ATP and viability as in (A2). When pyruvate is the only nutrient, ATP is even higher than in the presence of glucose alone (B1 compared to A1). Therefore, U87 cells can efficiently use pyruvate for ATP synthesis in the absence of glucose. A significant drop (*p* < 0.05) of ATP is observed when cells cultivated in pyruvate received carnosine (B2 compared to B1 and B5 compared to B3). Hence, carnosine does also inhibit mitochondrial ATP production. This inhibition is less pronounced than that on glycolysis and it is in agreement with recent observations of Shen and co- workers *et al*. These authors showed that carnosine is able to inhibit mitochondrial ATP-linked respiration in gastric cancer cells [1]. In addition, we have previously demonstrated that carnosine enhances the expression of pyruvate dehydrogenase kinase 4 (PDK4) [23], although, in further experiments (which were done in the presence of FBS and GlutaMax) we did not detect a change of pyruvate dehydrogenase activity under the influence of carnosine (unpublished data). Another result is the apparent incomplete inhibition of ATP production by CPI-613 when pyruvate is supplied, but glucose is absent (B3). This observation of the experiments presented in Figure 5 (and repetitions of it) is in contrast to the data presented in Figure 3 in which a concentration of 50 μM CPI-613 severely diminished the amount of ATP. It is tempting to speculate that this variation in the quantitative effect of CPI-613 on ATP is associated with the effect of CPI-613 on 2-oxoglutarate dehydrogenase. This would also disrupt mitochondrial metabolism [28], dependent on the current status of the oxidative defense systems of the cell. Moreover, the lipoate derivative CPI-613 could possibly also inhibit other mitochondrial enzyme complexes that require lipoate as coenzyme, e.g. the branched-chained alpha-ketoacid dehydrogenase complex [29]. However, these interpretations are speculative and CPI-613 at a concentration of 50 μM may simply not always completely block the pyruvate dehydrogenase activity. In fact, as demonstrated in the experiments of Zachar *et al*. [25], the IC50 for most of the cell lines tested was in the range between 120 and 280 μM. Despite the fact, their experiments are difficult to compare, as the authors used additional media supplements. In the presence of DNP (B4) the pyruvate supplied in the medium is not able to contribute to ATP production. Although, almost no ATP was determined in cell lysates (0.58 ± 0.06%; compared to pyruvate without inhibitor), up to 21% of the cells were still alive (Figure 7, yellow color in Figure 8, Table 1). Therefore, a strict correlation between ATP and viability has to be questioned. Nevertheless, full ATP production is important for 100% survival. It also has to be noted that the cells stained by Calcein AM had a severely changed morphology, indicating that they were also dying (data not shown). No cells are stained by Calcein AM in the presence of pyruvate and DNP when carnosine was added (B6 compared to B4). This indicates that carnosine also inhibits other biochemical pathways required for survival even in the absence of ATP production. This supports the notion that carnosine does in fact have pleiotropic effects [30]. The restoration of viability and ATP production in the experiments (C5) and (C6) whereby glucose and pyruvate are combined, demonstrates that exogenous pyruvate attenuates or even inhibits the effect of carnosine on glycolytic breakdown of glucose. We currently do not know at which step carnosine inhibits the glycolytic breakdown of glucose. This would require a detailed metabolomics study, and corresponding follow-up experiments, e.g. Western Blots, qRT-PCR and enzyme assays. Pyruvate could possibly restore NAD^+^ which is consumed in the glycolytic step from glyceraldehyde-3-phosphate to 1,3-bisphosphoglycerate. This presupposes that carnosine reduces the availability of NAD^+^. In that case, the concentrations of glycolytic triose phosphates (glyceraldehyde-3-phosphate and dihydroxyacetone-phosphate) will increase. In turn, the concentration of methylglyoxal will increase, which would result in protein and mitochondrial dysfunction [31]. This can explain the anti-neoplastic effects that cannot solely be attributed to the impairment of ATP production. However, this assumption is in contrast to the observation that carnosine inhibits the deleterious effects accompanied by enhanced production of methylglyoxal [32]. In addition, carnosine's abatement on senescence and increase of life-span in normal cells and healthy organisms argue against the assumption that carnosine has a general effect on the availability of NAD^+^ [33]. Hence, the hypothesis that carnosine reduces the availability of NAD^+^ will only make sense if the effect is tumor-cell specific. A second possibility is a reduced availability of pyruvate from the glycolytic breakdown of glucose under the influence of carnosine. This will also suppress the regeneration of NAD^+^ for ongoing glycolysis. It is known that carnosine does react with aldehydes [34], or as other histidine containing dipeptides, with carbonyl groups in general [35]. Therefore, it may react with one or several metabolites in the glycolytic pathway. However, as pyruvate attenuates the effect of carnosine even at a concentration ten times lower than that of the dipeptide, a competitive inhibition by pyruvate appears to be unlikely. Although the mechanisms by which pyruvate restores tumor cell viability in the presence of carnosine remain unknown, the hypothesis can be excluded that pyruvate inhibits the anti-neoplastic effect of carnosine by increased mitochondrial production of ATP [19].

Our data may appear to diminish the hope that carnosine could be used as an anti-cancer drug. Nevertheless, it should be taken into account that carnosine's effect on glycolysis is one aspect of its anti-neoplastic activity [36]. Results obtained with other cancer models demonstrated that carnosine does also influence signal transduction pathways such as HIF-1alpha [37] or Akt/mTOR/p70S6K [2]. In addition, effects on manganese superoxide dismutase and cyclin B1 expression have been reported [38]. At this point, it should be noted that previous experiments demonstrated that carnosine has long term effects on tumor cell growth *in vitro* [5] and *in vivo* [7] even in the presence of pyruvate and FBS. In conclusion, the results presented bear the challenge to enhance the anti-neoplastic effect of the dipeptide by an inhibition of mechanisms which attenuate its inhibition of glycolysis.

## MATERIALS AND METHODS

### Reagents

Unless otherwise stated, all chemicals were purchased from Sigma Aldrich (Taufkirchen, Germany) including the carnosine employed in this study (Cat.-Nr.: C9625/ Lot: BCBK4678V).

### Long-term cell culture

All cells were propagated in 250 ml culture flasks (Sarstedt AG & Co., Nümbrecht, Germany) using 10 ml of standard culture medium (SCM: DMEM / 4.5 g/l glucose, without pyruvate (Life Technologies, Darmstadt, Germany) supplemented with 10% fetal bovine serum (FBS superior, Biochrom, Berlin, Germany), 2 mM GlutaMax (Life Technologies) and Penicillin-Streptomycin (Life Technologies)) at 37°C and 5% CO_2_ in humidified air in an incubator. The human glioblastoma multiforme (GBM) cell lines T98G and U87 were obtained from ATCC (Manassas, USA) and the line LN405 from the German collection of microorganisms and cell cultures (DSMZ, Braunschweig, Germany). All cells were genotyped (Genolytic GmbH, Leipzig, Germany) and their identity confirmed.

### Cell culture experiments

For the experiments, cells were used from culture flasks at 70 to 90% confluency. Cells were first washed with 6 ml of Hanks (without Ca^2+^ / Mg^2+^: 137 mM NaCl; 5.4 mM KCl; 0.33 mM Na_2_HPO_4_ **×** 2 H_2_0; 0.44 mM KH_2_PO_4_; 2 mM Hepes (pH 7.4)) and then detached for 5 min in an incubator at 37°C using 2 ml Accutase (Life Technologies). Then, pre-warmed SCM (4 ml) was added and the cells were transferred to a centrifuge tube and collected by centrifugation (5 min; 125 × g). After aspiration of old medium, fresh SCM was added and 0.5x10^6^ cells were equally spread in 10 ml of SCM into new culture flasks (250 ml). After 72 hours cells were again harvested as described above and seeded at a density of 5000 cells per well in 96-well plates (μClear, Greiner Bio One, Frickenhausen, Germany) in SCM for 3 hours before they received fresh medium without glucose, galactose or pyruvate and without GlutaMax and FBS. Then cells were incubated for 20 or 24 hours (see individual experiment) before they received new medium with the supplements indicated in each experiment.

### Cell-based assays

For the determination of the amount of ATP in cell lysates the CellTiter-Glo Assay (CTG), for the determination of NAD(P)H production the CellTiter-Blue Assay (CTB) and for the determination of extracellular lactate dehydrogenase (LDH) activity (measuring loss of membrane integrity due to necrosis) the CytoTox-ONE Assay (all from Promega, Mannheim, Germany) were employed according to the instructions of the manufacturer and as described previously [39]. Briefly, the CellTiter-Glo Assay was performed by adding 100 μl of the CellTiter-Glo Assay reagent to the cells cultivated in 100 μl medium. Cells were lysed within the solution for 10 minutes and then luminescence was recorded using a Mithras LB 940 Multimode Microplate reader (Berthold Technologies, Bad Wildbad, Germany). The CellTiter-Blue assay was started by adding 20 μl of CellTiter-Blue reagent to the cells cultivated in 100 μl of medium. Ninety minutes later fluorescence was recorded at an excitation wavelength of 560 nm and an emission wavelength of 590 nm using a Spectra Max M5 reader (Molecular Devices, Biberach, Germany). At this point it may also be important to note that the presence of test compounds in the medium did not interfere with the assays (data not shown).

### Live cell imaging and cell counting

In order to determine viability at the microscopic level viable cells were stained with Calcein AM (Invitrogen, Darmstadt, Germany) and dead cells with propidium iodide (PI). Briefly, a mixture containing 20 μM Calcein AM and 120 μM propidium iodide (5 μl) was added to cells cultivated in 100 μl culture medium in a well of a 96-well plate (μClear black, Greiner Bio One). After an incubation of 10 minutes at 37°C, fluorescence images were captured using a Leica DM IRBE inverted fluorescence microscope (Leica microsystems, Wetzlar, Germany). Cells were counted using ImageJ scripts [40] and cell viability was determined by comparing the total cell number of cells to the number of living cells stained by Calcein AM and dead cells stained with propidium iodide.

### Statistical analysis

For the determination of mean and standard deviation as well as statistical significance by student's *t-test* (unpaired two-sample test with unequal variances) of single experiments the algorithms implemented in Excel (Microsoft, Richmond, USA) were used. Data obtained from more than one experiment were processed as followed: Raw data of each independent experiment was normalized by the sum of all corresponding data points [41]. Then, the mean and the standard deviation of all normalized data points for each condition were calculated and statistical significance determined by the algorithms implemented in Excel. For the calculation of inhibitory concentration (IC) data points were fitted to the Boltzmann function to plot a sigmoidal curve using Origin 8 (OriginLab Corporation, Northampton, USA).

## SUPPLEMENTARY FIGURES



## References

[1] Shen Y, Yang J, Li J, Shi X, Ouyang L, Tian Y, Lu J (2014). Carnosine inhibits the proliferation of human gastric cancer SGC-7901 cells through both of the mitochondrial respiration and glycolysis pathways. PLoS ONE.

[2] Zhang Z, Miao L, Wu X, Liu G, Peng Y, Xin X, Jiao B, Kong X (2014). Carnosine Inhibits the Proliferation of Human Gastric Carcinoma Cells by Retarding Akt/mTOR/p70S6K Signaling. J Cancer.

[3] Iovine B, Iannella ML, Nocella F, Pricolo MR, Baldi MR, Bevilacqua MA (2011). Carnosine inhibits KRas-mediated HCT-116 proliferation by affecting ATP and ROS production. Cancer Lett.

[4] Mikuła-Pietrasik J, Książek K (2016). L-Carnosine Prevents the Pro-cancerogenic Activity of Senescent Peritoneal Mesothelium Towards Ovarian Cancer Cells. Anticancer Res.

[5] Renner C, Seyffarth A, S Garcia de Arriba, Meixensberger J, Gebhardt R, Gaunitz F (2008). Carnosine Inhibits Growth of Cells Isolated from Human Glioblastoma Multiforme. Int J Pept Res Ther.

[6] Nagai K, Suda T (1986). Antineoplastic effects of carnosine and beta-alanine--physiological considerations of its antineoplastic effects. J Physiol Soc Jpn.

[7] Renner C, Zemitzsch N, Fuchs B, Geiger KD, Hermes M, Hengstler J, Gebhardt R, Meixensberger J, Gaunitz F (2010). Carnosine retards tumor growth in vivo in an NIH3T3-HER2/neu mouse model. Mol Cancer.

[8] Gaunitz F, Hipkiss AR (2012). Carnosine and cancer: a perspective. Amino Acids.

[9] Hipkiss AR, Gaunitz F (2014). Inhibition of tumour cell growth by carnosine: some possible mechanisms. Amino Acids.

[10] Gaunitz F, Oppermann H, Hipkiss AR, Preedy V. R (2015). Carnosine and Cancer. Imidazole Dipeptides.

[11] Renner C, Asperger A, Seyffarth A, Meixensberger J, Gebhardt R, Gaunitz F (2010). Carnosine inhibits ATP production in cells from malignant glioma. Neurol Res.

[12] Bensinger SJ, Christofk HR (2012). New aspects of the Warburg effect in cancer cell biology. Semin Cell Dev Biol.

[13] Koppenol WH, Bounds PL, Dang CV (2011). Otto Warburg‘s contributions to current concepts of cancer metabolism. Nat Rev Cancer.

[14] Frezza C, Gottlieb E (2009). Mitochondria in cancer: Not just innocent bystanders. Semin Cancer Biol.

[15] Christofk HR, MG Vander Heiden, Harris MH, Ramanathan A, Gerszten RE, Wei R, Fleming MD, Schreiber SL, Cantley LC (2008). The M2 splice isoform of pyruvate kinase is important for cancer metabolism and tumour growth. Nature.

[16] Sousa CM, Biancur DE, Wang X, Halbrook CJ, Sherman MH, Zhang L, Kremer D, Hwang RF, Witkiewicz AK, Ying H, Asara JM, Evans RM, Cantley LC (2016). Pancreatic stellate cells support tumour metabolism through autophagic alanine secretion. Nature.

[17] Hay N (2016). Reprogramming glucose metabolism in cancer: can it be exploited for cancer therapy?. Nat Rev Cancer.

[18] Asgari Y, Zabihinpour Z, Salehzadeh-Yazdi A, Schreiber F, Masoudi-Nejad A (2015). Alterations in cancer cell metabolism: the Warburg effect and metabolic adaptation. Genomics.

[19] Holliday R, McFarland GA (1996). Inhibition of the growth of transformed and neoplastic cells by the dipeptide carnosine. Br J Cancer.

[20] Ostrom QT, Gittleman H, Fulop J, Liu M, Blanda R, Kromer C, Wolinsky Y, Kruchko C, Barnholtz-Sloan JS (2015). CBTRUS Statistical Report: Primary Brain and Central Nervous System Tumors Diagnosed in the United States in 2008–2012. Neuro-Oncology.

[21] Louis DN, Perry A, Reifenberger G, von Deimling A, Figarella-Branger D, Cavenee WK, Ohgaki H, Wiestler OD, Kleihues P, Ellison DW (2016). The 2016 World Health Organization Classification of Tumors of the Central Nervous System: a summary. Acta Neuropathologica.

[22] Stupp R, Mason WP, van den Bent M. J, Weller M, Fisher B, Taphoorn MJB, Belanger K, Brandes AA, Marosi C, Bogdahn U, Curschmann J, Janzer RC, Ludwin SK (2005). Radiotherapy plus concomitant and adjuvant temozolomide for glioblastoma. New England Journal of Medicine.

[23] Letzien U, Oppermann H, Meixensberger J, Gaunitz F (2014). The antineoplastic effect of carnosine is accompanied by induction of PDK4 and can be mimicked by L-histidine. Amino Acids.

[24] Oppermann H, Ding Y, Sharma J, Bernd-Paetz M, Meixensberger J, Gaunitz F, Birkemeyer C (2016). Metabolic response of glioblastoma cells associated with glucose withdrawal and pyruvate substitution as revealed by GC-MS. Nutrition & Metabolism.

[25] Zachar Z, Marecek J, Maturo C, Gupta S, Stuart SD, Howell K, Schauble A, Lem J, Piramzadian A, Karnik S, Lee K, Rodriguez R, Shorr R (2011). Non-redox-active lipoate derivates disrupt cancer cell mitochondrial metabolism and are potent anticancer agents in vivo. J Mol Med (Berl).

[26] Loomis WF, Lipmann F (1948). Reversible inhibition of the coupling between phosphorylation and oxidation. J Biol Chem.

[27] Bhatt AN, Chauhan A, Khanna S, Rai Y, Singh S, Soni R, Kalra N, Dwarakanath BS (2015). Transient elevation of glycolysis confers radio-resistance by facilitating DNA repair in cells. BMC Cancer.

[28] Stuart SD, Schauble A, Gupta S, Kennedy AD, Keppler BR, Bingham PM, Zachar Z (2014). A strategically designed small molecule attacks alpha-ketoglutarate dehydrogenase in tumor cells through a redox process. Cancer Metab.

[29] Fujiwara K, Takeuchi S, Okamura-Ikeda K, Motokawa Y (2001). Purification, characterization, and cDNA cloning of lipoate-activating enzyme from bovine liver. J Biol Chem.

[30] Boldyrev AA, Aldini G, Derave W (2013). Physiology and pathophysiology of carnosine. Physiol Rev.

[31] Hipkiss AR (2010). Aging, Proteotoxicity, Mitochondria, Glycation, NAD and Carnosine: Possible Inter-Relationships and Resolution of the Oxygen Paradox. Front Aging Neurosci.

[32] Hipkiss AR, Chana H (1998). Carnosine protects proteins against methylglyoxal-mediated modifications. Biochem Biophys Res Commun.

[33] Hipkiss AR (2010). NAD(+) and metabolic regulation of age-related proteoxicity: A possible role for methylglyoxal?. Exp Gerontol.

[34] Xie Z, Baba SP, Sweeney BR, Barski OA (2013). Detoxification of aldehydes by histidine-containing dipeptides: From chemistry to clinical implications. Enzymology and Molecular Biology of Carbonyl Metabolism.

[35] Vistoli G, Colzani M, Mazzolari A, Maddis DD, Grazioso G, Pedretti A, Carini M, Aldini G (2016). Computational approaches in the rational design of improved carbonyl quenchers: focus on histidine containing dipeptides. Future Med Chem.

[36] Hipkiss AR, Gaunitz F (2013). Inhibition of tumour cell growth by carnosine: some possible mechanisms. Amino Acids.

[37] Iovine B, Guardia F, Irace C, Bevilacqua MA (2016). l-carnosine dipeptide overcomes acquired resistance to 5-fluorouracil in HT29 human colon cancer cells via downregulation of HIF1-alpha and induction of apoptosis. Biochimie.

[38] Rybakova YS, Kalen AL, Eckers JC, Fedorova TN, Goswami PC, Sarsour EH (2015). Increased manganese superoxide dismutase and cyclin B1 expression in carnosine-induced inhibition of glioblastoma cell proliferation. Biomed Khim.

[39] Gaunitz F, Heise K (2003). HTS compatible assay for antioxidative agents using primary cultured hepatocytes. Assay.Drug Dev.Technol.

[40] Schneider CA, Rasband WS, Eliceiri KW (2012). NIH Image to ImageJ: 25 years of image analysis. Nat Methods.

[41] Degasperi A, Birtwistle MR, Volinsky N, Rauch J, Kolch W, Kholodenko BN (2014). Evaluating strategies to normalise biological replicates of Western blot data. PLoS ONE.

