# Supplementary material for: Pyruvate attenuates the anti-neoplastic effect of carnosine independently from oxidative phosphorylation
Source: Oncotarget. 2016 Nov 3;7(52):85848–60. doi: 10.18632/oncotarget.13039 (PMC5349879; doi:10.18632/oncotarget.13039)
Supplement: Supplementary file 1 [file oncotarget-07-85848-s001.pdf]

Pyruvate attenuates the anti-neoplastic effect of carnosine independently from oxidative phosphorylation

Supplementary Materials

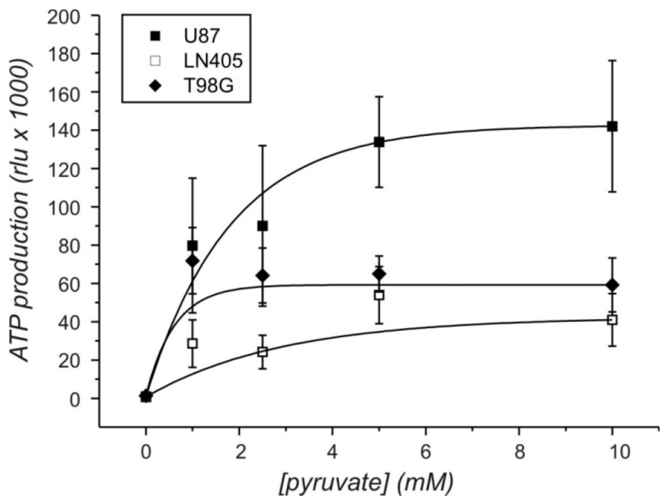

Supplementary Figure S1: ATP production under the influence of pyruvate in glioblastoma cells treated with carnosine. Cells from the lines U87, LN405 and T98G were seeded at a density of 5000 cells per well in 96-well microplates and received medium without a carbon source, GlutaMAX and FBS for 20 hours. Then fresh medium was added with different concentrations of pyruvate (1 mM, 2.5 mM, 5 mM and 10 mM) in the presence of 50 mM carnosine. 24 hours later ATP production was determined as relative light units using the CellTiter-Glo Assay. Results are represented as mean and standard deviation of measurement of 6 independent wells. Data points have been fitted using an exponential function.

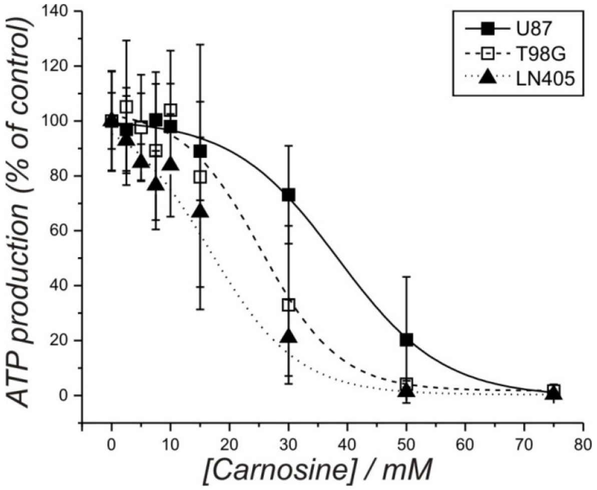

Supplementary Figure S2: ATP production of cells from the line U87, T98G and LN405 under the influence of different concentrations of carnosine. Cells from the line U87, T98G and LN405 were seeded at a density of 5000 cells per well in 96-well microplates. After 20 hours cells received fresh medium with different concentrations of carnosine (0 mM, 2.5 mM, 5 mM, 7.5 mM, 10 mM, 15 mM , 30 mM, 50 mM, 75 mM) and glucose (25 mM). 24 hours later ATP production was determined using the CellTiter-Glo assay and the half maximal inhibitory concentration (IC50) as well as the IC20 and the IC80 were determined by fitting the data into a sigmoidal curve by the Boltzmann Function (see table). Results are represented as mean and standard deviation of 6 wells for each condition normalized to viability of cells cultivated in the presence of glucose without carnosine (set as 100%).

Cell/Correlation Coefficient	IC20	IC50	IC80
U87/R^2 = 0.999	25.0 mM	38.3 mM	50.7 mM
T98G/R^2 = 0.995	16.2 mM	25.4 mM	34.5 mM
LN405/R^2 = 0.995	6.4 mM	16.7 mM	26.0 mM
